# Distinct Interactions with Cellular E-Cadherin of the Two Virulent Metalloproteinases Encoded by a *Bacteroides fragilis* Pathogenicity Island

**DOI:** 10.1371/journal.pone.0113896

**Published:** 2014-11-20

**Authors:** Albert G. Remacle, Sergey A. Shiryaev, Alex Y. Strongin

**Affiliations:** Infectious and Inflammatory Disease Center, Sanford-Burnham Medical Research Institute, La Jolla, California, United States of America; University of Ulster, United Kingdom

## Abstract

*Bacteroides fragilis* causes the majority of Gram-negative anaerobic infections in the humans. The presence of a short, 6-kb, pathogenicity island in the genome is linked to enterotoxigenic *B. fragilis* (ETBF). The role of the enterotoxin in *B. fragilis* virulence, however, remains to be determined, as the majority of clinical isolates lack ETBF genes and healthy individuals carry enterotoxin-positive *B. fragilis*. The island encodes secretory metalloproteinase II (MPII) and one of three homologous enterotoxigenic fragilysin isoenzymes (FRA; also termed *B. fragilis* toxin or BFT). The secretory metalloproteinases expressed from the genes on the *B. fragilis* pathogenicity island may have pathological importance within the gut, not linked to diarrhea. MPII and FRA are counter-transcribed in the bacterial genome, implying that regardless of their structural similarity and overlapping cleavage preferences these proteases perform distinct and highly specialized functions in the course of *B. fragilis* infection. The earlier data by us and others have demonstrated that FRA cleaves cellular E-cadherin, an important adherens junction protein, and weakens cell-to-cell contacts. Using E-cadherin-positive and E-cadherin–deficient cancer cells, and the immunostaining, direct cell binding and pull-down approaches, we, however, demonstrated that MPII *via* its catalytic domain efficiently binds, rather than cleaves, E-cadherin. According to our results, E-cadherin is an adherens junction cellular receptor, rather than a proteolytic target, of the *B. fragilis* secretory MPII enzyme. As a result of the combined FRA and MPII proteolysis, cell-to-cell contacts and adherens junctions are likely to weaken further.

## Introduction

The Gram-negative, anaerobic *Bacteroides* is the most abundant genus in the human microbiome. Commensal *Bacteroides* strains are critical to systemic and mucosal immunity and host nutrition [1]. Enterotoxigenic *B. fragilis* strains are implicated in chronic inflammation (e.g., inflammatory diarrhea and ulcerative colitis) and are linked to the induction of colon tumorigenesis in a murine model *via* stimulation of a selective pro-carcinogenic intra-colonic Th17 immune response [2]. In *B. fragilis*, a pathogenicity island contains two distinct metalloproteinase genes encoding metalloproteinase II (MPII) and fragilysin (FRA; also called *B. fragilis* toxin or BFT) [3]–[8]. FRAs exist in three homologous isoforms (FRA1, 2 and 3) with sequence identities of over 95%, while the sequence identity between FRAs and MPII is ∼25%. Both FRA and MPII are secretory metalloproteinases with a zinc-binding HEXXHXXGXXH motif and a characteristic Met-turn [9]–[11] in their catalytic domain that has a classic “metzincin” fold typical of eukaryotic matrix metalloproteases. The crystal structure of FRA3 was reported earlier [10]. Recently, we have determined the X-ray 2.1 Å structure of MPII [11]. Despite the low sequence identity, the three-dimensional structures are closely related. They comprise a large N-terminal regulatory domain unrelated to any known folds, followed by a C-terminal catalytic domain.

FRAs and MPII are counter-transcribed by *B. fragilis* in which MPII is poorly expressed *in vitro* under growth conditions favoring expression of FRAs. This unconventional transcription regulation suggests a distinct function of these proteinases *in vivo* [12], [13]. In agreement, our earlier data reveal that MPII is a *bona fide* protease that is structurally homologous to FRA, but has the distinct, albeit partially overlapping, substrate cleavage preferences when compared with FRA [11], [14].

In this paper, we describe our attempts to elucidate the likely biochemical mechanisms involved in the interaction of MPII with the host cell. We determined that the catalytic domain of MPII directly binds to, but does not cleave, the specific and abundant cell surface protein, E-cadherin. The latter is a main component of the cell-cell adhesion junctions, which play a principal role in maintaining normal epithelial cell morphology. In contrast and in agreement with the results by others [15], [16], FRA directly cleaves, rather than binds to, E-cadherin. Overall, MPII and FRA3 likely perform distinct functions in the course of *B. fragilis* infection. Our data also suggest that following its binding to E-cadherin, MPII is capable of performing the focused proteolysis of the cell host proteins. This is in contrast with FRA that appears cleaving cellular E-cadherin in a soluble proteinase form [13], [15], [16]. As a result, adherens junctions and cell-to-cell contacts are likely to weaken further. The identification of the target of MPII proteolysis is now in progress.

## Materials and Methods

### General reagents and antibodies

All reagents were purchased from Sigma-Aldrich (St. Louis, MO) unless indicated otherwise. Mammary epithelial cell growth medium (MEGM) was from Life Technologies (Grand Island, NY). McCoy's 5A and Dulbecco's modified Eagle's media (DMEM), sulfosuccinimidyl-2-(biotin-amido) ethyl-1,3-dithiopropionate (EZ-Link sulfo-NHS-SS-biotin) and a SuperSignal West Dura Extended Duration Substrate kit were from Thermo Fisher Scientific (Waltham, MA). Protein A-agarose beads, a broad-spectrum hydroxamate metalloproteinase inhibitor (GM6001) and a TMB/M substrate were from EMD Millipore (Temecula, CA). A murine monoclonal antibody (# 610181) to the 735-883 C-terminal cytoplasmic portion of E-cadherin was from BD Transduction Laboratories (San Diego, CA). A murine CD44 monoclonal (# 3570) and a rabbit Hisx6-tag polyclonal antibody (# 2365) were from Cell Signaling (Danvers, MA). The horseradish peroxidase (HRP)-conjugated donkey anti-mouse and anti-rabbit IgGs were from Jackson ImmunoResearch Laboratories (West Grove, PA).

### Cells

All cell lines were originally obtained from the American Type Culture Collection (Manassas, VA). Human mammary epithelial 184B5 cells were maintained in MEGM. Human colorectal carcinoma HTC116, HT29 and HT29/C1 cells (the latter was a kind gift of Dr. Cynthia L. Sears, The John Hopkins University, Baltimore, MD) were grown in McCoy's 5A medium. Human fibrosarcoma HT1080, breast carcinoma MCF-7 and glioma U251 cells were maintained in DMEM. All cell lines were routinely grown in the recommended cell growth medium supplemented with 10% fetal bovine serum and gentamicin (10 µg/ml).

### Recombinant proteins

**Figure 1:**
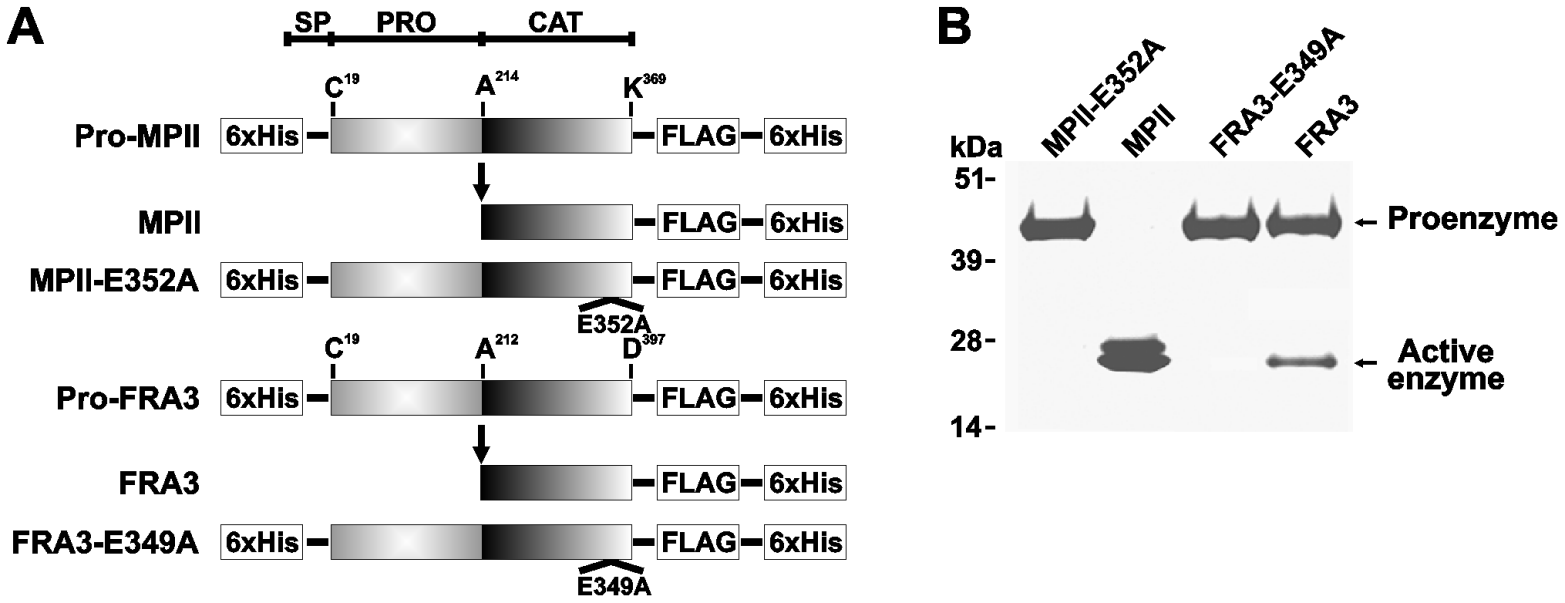
MPII and FRA3 recombinant constructs. **A**, the arrow indicates the position of self-conversion of the proenzyme into the fully active enzyme. The constructs were tagged with the Hisx6 and FLAG tags. E349A and E352A indicate the base substitution sites that transform the wild-type FRA3 and MPII into the catalytically inactive constructs of FRA3 and MPII, respectively. As a result of these inactivating mutations, Ala substitutes for the essential active site Glu residue. SP, signal peptide; PRO, prodomain; CAT, catalytic domain. **B**, SDS-gel electrophoresis of the purified MPII and FRA3 constructs followed by Coomassie staining.

The catalytically active MPII and FRA3 constructs, and the MPII-E252A and FRA3-E349A catalytically inactive mutants were obtained earlier [11], [14]. The constructs contained the N-terminal Hisx6-tag and also the C-terminal FLAG-tag directly followed by the additional Hisx6-tag (Fig. 1A). To increase the yield, the recombinant constructs were expressed in *E. coli* BL21 CodonPlus RIPL cells (Agilent Technologies, Santa Clara, CA) and then purified from the soluble fraction of *E. coli* lysate using metal-chelating chromatography. The purified samples were combined, dialyzed against 20 mM Tris-HCl, pH 8.0, containing 150 mM NaCl. The purified material was kept at −80°C until use. The purity of the material was tested by SDS gel-electrophoresis in a 12% NuPAGE-MOPS gel (Life Technologies, Grand Island, NY) followed by Coomassie staining and by Western blotting with the Flag-tag antibody. The samples with the purity level over 95% were used in our experiments.

### Biotinylation of MPII and FRA3

MPII, MPII-E352A, FRA3 and FRA3-E349A (50–100 µl at 1–2 mg/ml each) were labeled for 30 min on ice at a 1∶20 protein-biotin molar ratio using EZ-Link sulfo-NHS-SS-biotin. Excess biotin was removed using a desalting spin-column (Thermo Scientific).

### Binding of MPII and FRA3 to the cells, and 2-mercaptoethane sulfonic acid (MESNA) treatment

184B5, MCF-7, U251, HT1080, HT29, HT29/C1 and HCT116 cells were grown in the respective medium-10% FBS in wells of a 12-well plate until a 80–90% confluence level was reached. Cells were then washed with PBS and co-incubated for 3 h at 37°C with biotin-labeled MPII (b-MPII, 5 µg/ml), MPII-E352A (b-MPII-E352A, 0.1–10 µg/ml) or FRA3-E349A (b-FRA3-E349A, 10 µg/ml) in 0.7 ml of the respective serum-free medium. Cells were next extensively washed with ice-cold PBS, and lysed for 1 h at 4°C using 50 mM OG buffer [50 mM octyl-β-D-glucopyranoside in TBS, pH 7.4, supplemented with 1 mM phenylmethylsulfonyl fluoride, 10 mM EDTA, 10 µM GM6001 and a proteinase inhibitor cocktail (104 mM AEBSF, 80 µM aprotinin, 4 mM bestatin, 1.4 mM E-64, 2 mM leupeptin and 1.5 mM pepstatin A)]. Insoluble material was removed by centrifugation (14,000× *g*; 15 min). The supernatant aliquots (10 µg total protein) were separated by reducing or non-reducing SDS-gel electrophoresis in a 4–12% gradient NuPAGE-MOPS gel and analyzed by Western blotting with either HRP-conjugated ExtrAvidin (dilution 1∶10,000) or the Hisx6-tag antibody (dilution 1∶1,000) and E-cadherin antibody (dilution 1∶3,000) followed by the HRP-conjugated species-specific secondary antibody (dilution 1∶5,000) and either a SuperSignal West Dura Extended Duration Substrate kit or a TMB/M substrate. Where indicated, cells were treated for 25 min on ice in Sorenson phosphate buffer (14.7 mM KH_2_PO_4_, 2 mM Na_2_HPO_4_, 120 mM sorbitol, pH 8.4) containing membrane-impermeable MESNA reducing agent (150 mM) to remove the residual cell surface biotin [17]. In some additional experiments, cells were co-incubated for 1 h at 37°C with FRA3 (500 ng/ml), FRA3-E349A (10 µg/ml) or b-FRA3-E349A (10 µg/ml) and then additionally co-incubated with b-MPII-E352A (7.5 µg/ml) for 3 h at 37°C.

### Proteolysis of E-cadherin

184B5, MCF-7, U251, HT1080, HT29 and HCT116 cells were grown in wells of a 12-well plate in the respective medium-10% FBS to reach a 80–90% confluence level. After washing with PBS, cells were left intact or co-incubated for 3 h at 37°C with MPII (10 µg/ml), MPII-E352A (10 µg/ml), FRA3 (500 ng/ml) or FRA3-E349A (10 µg/ml) individually or jointly in 0.7 ml of the respective serum-free medium. Cells were next washed with ice-cold PBS, and lysed using 50 mM OG buffer as described above. The supernatant aliquots (10 µg total protein) were separated by SDS-gel electrophoresis in a reducing 4–12% gradient NuPAGE-MOPS gel and analyzed by Western blotting with the E-cadherin antibody followed by the secondary donkey anti-mouse HRP-conjugated antibody and a TMB/M substrate.

### Immunofluorescence microscopy

HT29 cells (25–75% confluence) grown on a poly-D-lysine coated coverslip were washed with serum-free McCoy's 5A medium containing 20 mM HEPES, pH 7.0, and 0.2% BSA, incubated for 30 min at 37°C, and then co-incubated for 3 h at 37°C with MPII, MPII-E352A, FRA3 or FRA3-E349A (5 µg/ml, each) in the same medium. Cells were next fixed for 16 min using 4% paraformaldehyde and then either left intact or permeabilized for 4 min using 0.1% Triton X-100 in 4% paraformaldehyde. After blocking the coverslips for 1 h in 10% BSA, cells were stained for 16–18 h at 4°C using the polyclonal Hisx6-tag antibody (dilution 1∶200), the monoclonal FLAG M2 antibody (dilution 1∶500) or the monoclonal E-cadherin antibody (dilution 1∶100) in PBS-1% BSA followed by incubation for 1 h with the secondary species-specific Alexa Fluor-488-conjugated goat anti-rabbit IgG or Alexa Fluor-594-conjugated goat anti-mouse IgG (dilution 1∶200, each). The slides were mounted in Vectashield DAPI-containing medium and analyzed using an Olympus BX51 fluorescence microscope equipped with a MagnaFire digital camera (Olympus). Cells were also observed using a bright-field microscope. Where indicated, U251 cells were co-incubated with the MPII and FRA3 constructs in serum-free DMEM supplemented with 20 mM HEPES, pH 7.0 and 0.2% BSA, instead of McCoy's 5A medium.

### Immunoprecipitation of E-cadherin and MPII-E352A

The whole cell extracts obtained in 50 mM OG buffer were diluted 2.5-fold to reduce the detergent concentration. Extract aliquots (2 mg total protein in 20 mM OG buffer each) were pre-cleared for 2 h at 4°C using the anti-FLAG M2 affinity gel (50 µl, 50% slurry). The samples were then left intact or co-incubated for 2 h at 4°C with either MPII-E352A or FRA3-E349A (2 µg each). MPII-E352A and FRA3-E349A were pulled-down for 16–18 h at 4°C using the anti-FLAG M2 affinity gel (50 µl, 50% slurry). Following extensive washes in 20 mM OG buffer, followed by 2 washes in TBS, pH 7.4, the bound material was eluted from the beads using FLAG peptide (0.3 mg/ml, 30 µl, 1 h, 4°C). The samples were separated by SDS-gel electrophoresis in a 4–12% gradient NuPAGE-MOPS gel and analyzed by Western blotting with the monoclonal E-cadherin and CD44 antibodies (dilution 1∶3,000 and 1∶2,000, respectively) followed by the secondary HRP-conjugated donkey anti-mouse antibody (dilution 1∶5,000) and a SuperSignal West Dura Extended Duration Substrate kit.

## Results

### Recombinant MPII and FRA3 constructs

To explore the biochemical properties of MPII and FRA3 (a representative of the three FRA isoenzymes), we expressed the full-length MPII and FRA3 genes in *E. coli*. The constructs were flanked by an N-terminal and C-terminal Hisx6-tag and, additionally, by a C-terminal FLAG-tag (Fig. 1A). The constructs were purified from lysates of *E. coli*. In agreement with our earlier observations [11], [14], the MPII proenzyme was readily self-activated during purification and transformed into the proteolytically active MPII enzyme. In turn, the FRA3 proform was more stable and only a fraction of the active enzyme accumulated in the FRA3 samples. To obtain the full-length stable proenzymes lacking self-activation, the catalytically inactive MPII-E352A and FRA3-E349A mutants, in which the catalytically essential Glu residue of the active site was mutated into Ala, were also constructed, expressed and purified (Fig. 1B).

### Direct binding of MPII to cell surfaces

**Figure 2:**
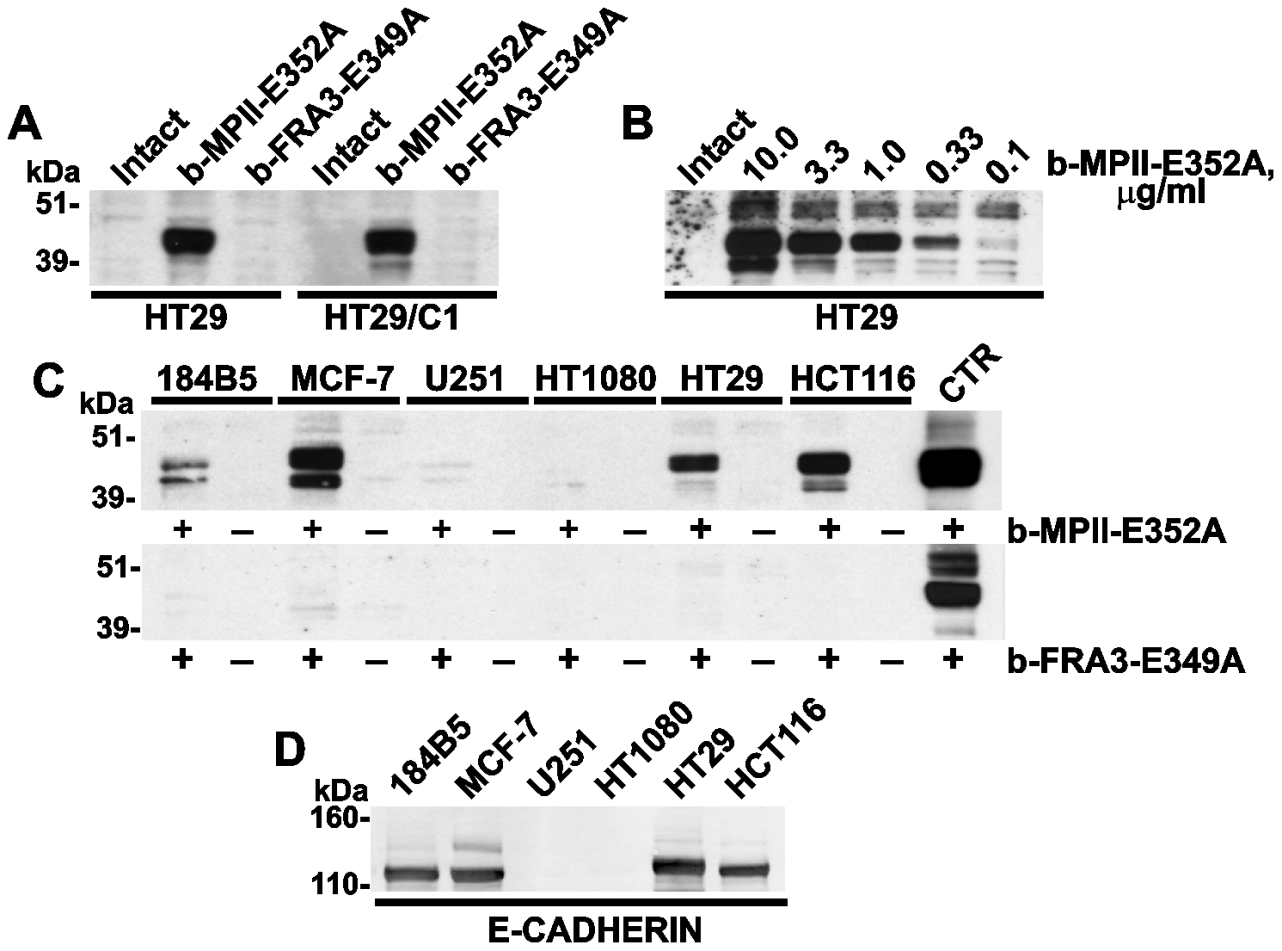
MPII, but not FRA3, binds to the surface of the E-cadherin-expressing cells. **A**, HT29 and HT29/C1 cells were left intact or incubated for 3 h at 37°C with b-MPII-E352A or b-FRA3-E349A (10 µg/ml each). **B**, HT29 cells were left intact or incubated for 3 h at 37°C with the indicated concentrations of b-MPII-E352A. **C**, 185B5, MCF-7, U251, HT1080, HT29 and HCT116 cells were incubated for 3 h at 37°C with b-MPII-E352A or b-FRA3-E349A (10 µg/ml each). In A, B and C, cell lysates were analyzed by Western blotting with ExtrAvidin-HRP. Control (CTR), b-MPII-E352A or b-FRA3-E349A (10 ng) in whole cell extract of HT29 cells (10 µg total protein). **D**, E-cadherin expression in 185B5, MCF-7, U251, HT1080, HT29 and HCT-116 cells. Cell lysates were analyzed by Western blotting with the E-cadherin antibody.

Human colonic epithelial HT29 cells and the HT29/C1 cell sub-clone that was specially developed to identify toxigenic *B. fragilis* strains [18], were allowed to bind the biotin-labeled purified MPII-E352A (b-MPII-E352A) and FRA-E349A (b-FRA-E349A) constructs. Following co-incubation of HT29 and HT29/C1 cells with b-MPII-E352A and b-FRA-E349A, cells were extensively washed to remove the unbound material and lysed. To detect the bound biotin-labeled proteinases, the lysate aliquots were then analyzed by Western blotting with ExtrAvidin. The results showed that b-MPII-E352A bound with equal efficiency to the cell surfaces of both cell types whereas no binding of b-FRA-E349A was observed (Fig. 2A).

In order to test if there was a concentration-dependence of the b-MPII-E352A binding to HT29 cells, HT29 cells (1×10^6^) were co-incubated with the increasing concentrations of b-MPII-E352A and the bound biotin-labeled material was determined using Western blotting of the cell lysate aliquots. The levels of the bound b-MPII-E352A in cell extracts were directly proportional to the concentrations of soluble b-MPII-E352A we added to the cells. Conversely, the high level of the bound b-MPII-E352A detected in the cell sample could be a result of the correspondingly high level of the MPII's putative cell receptor that should be present in a range of million(s) of molecules per cell (Fig. 2B).

### MPII binding correlates with the levels of cellular E-cadherin

To shed more light on the origin of the MPII putative receptor, we investigated the binding efficiency of b-MPII-E352A with the cell surface of cancer cells of the distinct origin, including normal mammary epithelial 184B5 cells, breast carcinoma MCF-7, glioma U251, fibrosarcoma HT1080,and colorectal carcinoma HT29 and HCT116 cells. (Fig. 2C). There was no binding of b-FRA-E349A in any cells we tested. In contrast, the levels of bound b-MPII-E352A were readily observed in the cells of epithelial origin, including 184B5 and HT29 cells. The bound b-MPII-E352A was especially high in MCF-7 and HCT116 cells. In non-epithelial cells such as U251 and HT1080 cells no binding of b-MPII-E352A was recorded. Intriguingly, these epithelial cells are known to express high levels of the E-cadherin tight junction protein [19], [20] while highly migratory, invasive U251 and HT1080 cells express minute levels of E-cadherin. In agreement with this, analysis of the cell samples using Western blotting with the E-cadherin antibody confirmed the expression of E-cadherin in 184B5, MCF-7, HT29 and HCT116 cells. E-cadherin was not detected in HT1080 and U251 cell samples (Fig. 2D). The binding of MPII to MCF-7 and HT29 cells, however, did not directly correlate with the E-cadherin levels we recorded in these cells. The results of multiple blots were highly reproducible. A representative blot of each experiment is shown in the figures.

### The catalytic domain of MPII binds cell surfaces

**Figure 3:**
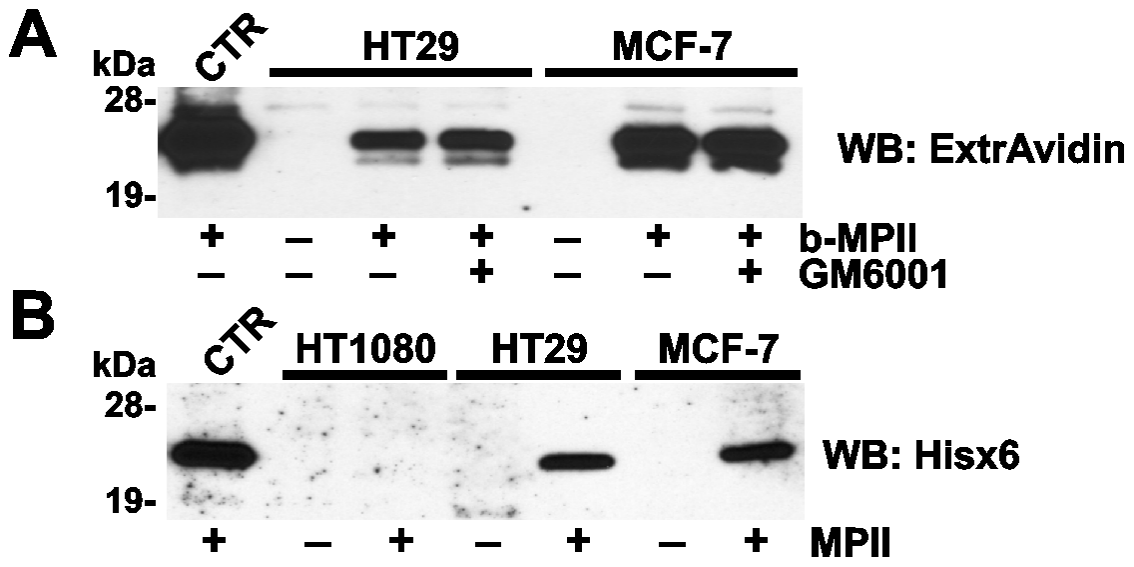
MPII interacts with cell surfaces through its catalytic domain. **A**, HT29 and MCF-7 cells were left intact or incubated for 3 h at 37°C with b-MPII (10 µg/ml). Where indicated, cells were incubated in the presence of GM6001 (25 µM). Control (CTR), purified b-MPII (10 ng) was added to the whole cell extract of HT29 cells (total protein, 10 µg). **B**, HT1080, HT29 and MCF-7 cells were left intact or incubated for 3 h at 37°C with MPII (10 µg/ml). Control (CTR), MPII (10 ng) was added to the whole cell extract of HT29 cells (total protein, 10 µg). Cell lysates were analyzed by Western blotting with ExtrAvidin-HRP and the Hisx6-tag antibody in A and B, respectively.

In order to determine whether the catalytic activity was required for the association of MPII with its putative cell receptor, HT29 and MCF-7 cells were co-incubated with the biotin-labeled MPII self-activated enzyme (b-MPII) in the presence or absence of the excess of GM6001 (25 µM), a 30 nM range inhibitor of MPII [14]. No effect of GM6001 was observed on the binding of b-MPII to the cells, suggesting that the proteolytic activity is not a factor in the MPII-receptor binding process (Fig. 3A). Our data also indicate that the catalytic domain alone is sufficient for the efficient binding of MPII to its cell receptor and, conversely, that the absence of the prodomain does not affect the binding of MPII to the cells.

In order to test these observations further, HT1080, MCF-7 and HT29 cells were co-incubated with the intact MPII self-activated enzyme. Following cell lysis, the bound MPII was detected in the cell lysate aliquots using Western blotting with the Hisx6-tag antibody. In agreement with our other data, bound MPII was readily detected in E-cadherin-rich HT29 and MCF-7 cells, but not in E-cadherin-deficient HT1080 cells (Fig. 3B).

### MPII-receptor interactions

**Figure 4:**
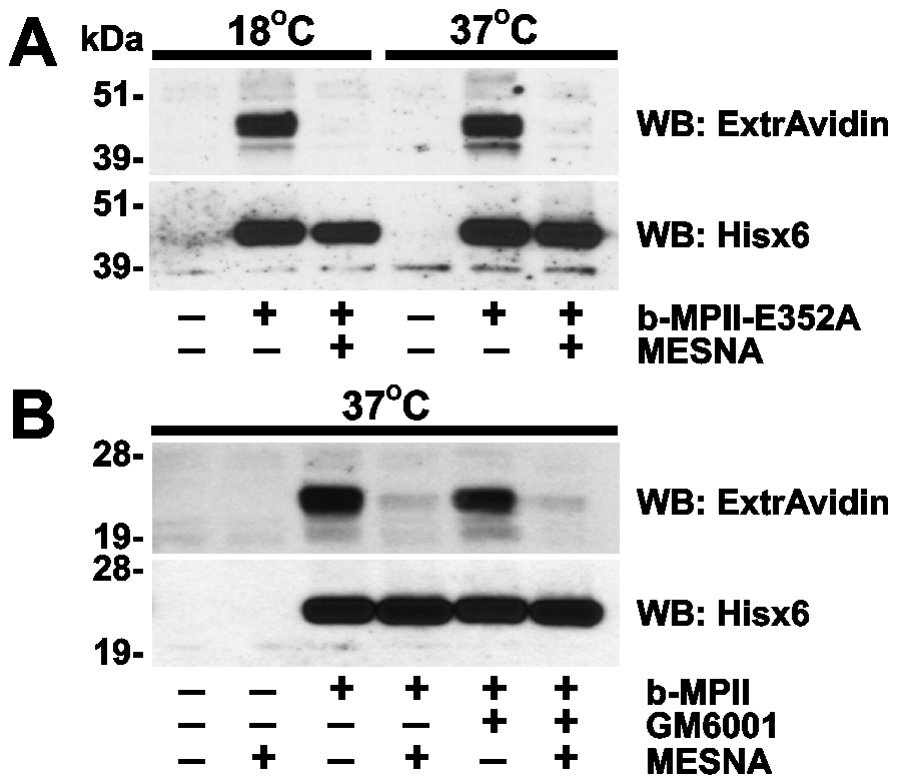
MPII is not efficiently internalized by the cells. **A**, HT29 cells were left intact or incubated for 3 h at 18°C or 37°C with b-MPII-E352A (10 µg/ml). B, HT29 cells were incubated for 3 h at 37°C with b-MPII (10 µg/ml). In A and B, where indicated, MESNA or GM6001 (25 µM) was added to the cells. Cell lysates were analyzed by Western blotting with ExtrAvidin-HRP or the Hisx6-tag antibody.

In order to further elucidate the interaction between MPII and its cell receptor, we determined if the MPII-receptor complex was efficiently internalized from the plasma membrane and into the cell compartment following the binding to the cell-surface receptor. Because temperature affects the efficiency of protein uptake by the cells, we co-incubated HT29 cells with the b-MPII-E352A proenzyme or the b-MPII enzyme constructs at 18°C or 37°C (Fig. 4A, B). Following the incubation and washings to remove the unbound protease, the cells were treated with MESNA. The latter cleaves off EZ-Link sulfo-NHS-SS-biotin from the exposed biotinylated proteins but not from the internalized proteins that are protected from membrane-impermeable MESNA by the plasma membrane [17]. Following MESNA treatment, the cells were lysed. To detect cell-associated b-MPII-E352A and b-MPII, cell lysates were analyzed using Western blotting with both ExtrAvidin and the Hisx6-tag antibody. Our experiments demonstrated that MESNA treatment efficiently removed biotin from the cell-associated protease constructs suggesting that both b-MPII-E352A and b-MPII are not readily internalized following their cell receptor binding. These internalization parameters correlate well with the features of E-cadherin, an adherens junction protein that is continuously present in the stable cell-to-cell contacts [19].

### Immunostaining of cells

**Figure 5:**
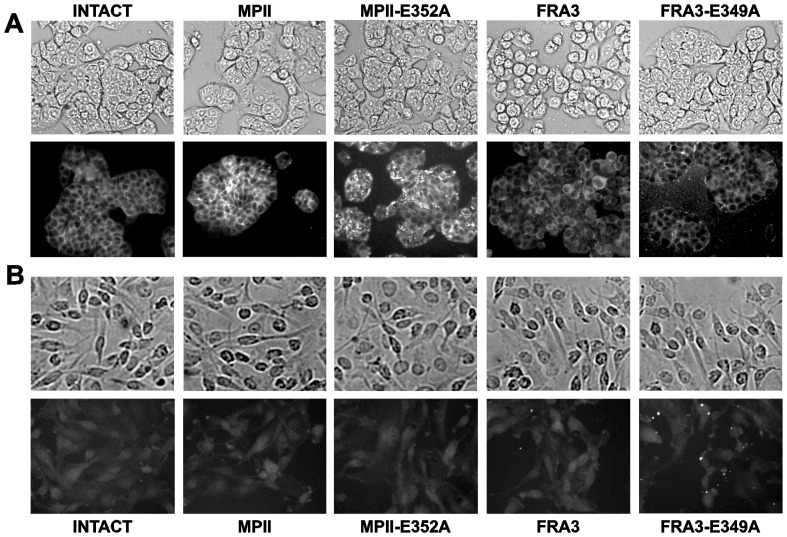
Immunostaining of cell-bound MPII. **A**, HT29 cells. **B**, U251 cells. Cells were left intact or incubated for 3 h at 37°C with MPII, MPII-E352A, FRA3 or FRA3-E349A (5 µg/ml, each). Cells were fixed, permeabilized and stained with the FLAG M2 antibody. Cell morphology was also observed using a bright-field microscope.

To visualize the interactions further, we co-incubated both HT29 and U251 cells with active MPII and FRA3, and also with the inactive MPII-E352A and FRA3-E349A mutant constructs. Following incubation cell morphology was observed using a bright-field microscope. In addition, cells were fixed and stained with the FLAG M2 antibody that recognizes the C-terminal FLAG-tag in the proteinase constructs. In agreement with our earlier results [11], MPII, MPII-E352A and FRA3-E349A did not affect cell morphology in HT29 and U251 cells. Because of FRA3 proteolysis of E-cadherin and weakening of cell-to-cell contacts, there was a drastic change in morphology in E-cadherin-rich HT29 cells but not in E-cadherin-deficient U251 cells (Fig. 5A, B). The results of our multiple additional immunostainings were highly similar. These results are consistent with the earlier data by us and others [8], [11], [13], [15], [16], [21]. In turn, there was an intensive MPII-FLAG immunoreactivity in HT29 cells co-incubated with MPII or MPII-E352A, but not with FRA3 or FRA3-E349A. No noticeable interactions of either MPII or MPII-E352A were recorded in U-251 cells. The staining pattern of HT29 cells with the MPII enzyme was less punctate compared with that of the MPII-E352A inactivated proform construct.

### FRA3 cleaves E-cadherin and reduces MPII binding to the cells

**Figure 6:**
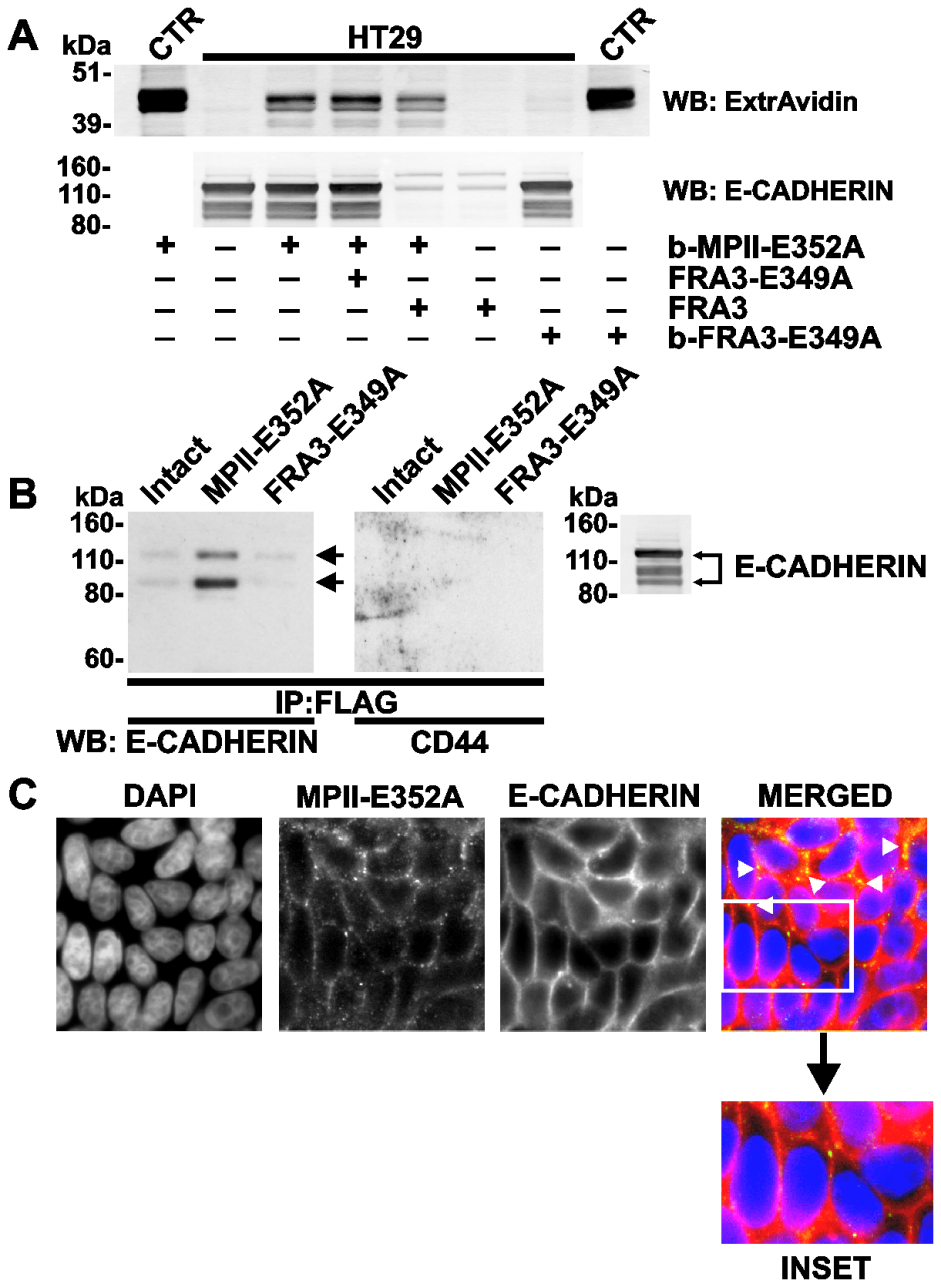
Co-immunoprecipitation and co-localization of E-cadherin with MPII. **A**, HT29 cells were left intact or incubated for 1 h at 37°C with FRA3 (500 ng/ml), FRA3-E349A and b-FRA3-E349A (10 µg/ml both). Cells were incubated for an additional 3 h at 37°C in the presence or absence of b-MPII-E352A (5 µg/ml). Cell lysates were analyzed by Western blotting with ExtrAvidin-HRP and the E-cadherin antibody. Control (CTR), b-MPII-E352A or b-FRA3-E349A (10 ng each) was added to the whole cell extract of the cells (10 µg total protein). **B**, Whole HT29 cell extract (2 mg total protein) were left intact or co-incubated with MPII-E352A or FRA3-E349A (2.5 µg each). MPII-E352A and FRA3-E349A were then immunoprecipitated (IP) using the FLAG M2 antibody-beads. The precipitates were analyzed by Western blotting with the E-cadherin and CD44 antibodies. *Right*, the whole HT29 cell extract aliquot (10 µg total protein) was analyzed by Western blotting with the E-cadherin antibody. Arrows point to E-cadherin co-precipitated with MPII. **C**, HT29 cells were incubated for 3 h at 37°C with MPII-E352A (5 µg/ml) and stained with the Hisx6 and E-cadherin antibodies (green and red, respectively). The nuclei were stained with DAPI (blue). The arrows point to the cell-cell junction regions in which MPII and E-cadherin co-localize. Inset, the enlarged area highlights the nuclear (DAPI) and E-cadherin membrane staining.

Because FRA3 proteolysis decreases the cellular E-cadherin levels [11], [15], we tested whether the FRA3-induced decrease in the levels of E-cadherin would cause the less efficient binding of MPII to the cells. Thus, HT29 cells were left intact or co-incubated with active FRA3 or the inactive FRA3-E349A and b-FRA3-E349A purified proteins. The cells were then further co-incubated with b-MPII-E352A. After washings, the cells were lysed. We next used cell lysate aliquots to determine the residual levels of cellular E-cadherin and also the levels of cell-bound b-MPII-E352A by Western blotting with the E-cadherin antibody and ExtrAvidin, respectively (Fig. 6A). Co-incubation of the cells with active FRA3, but not with intact or biotin-labeled inactive FRA3-E349A, significantly reduced the levels of E-cadherin in HT29 cells. The reduction in cellular E-cadherin correlated well with the less efficient binding of b-MPII-E352A to HT29 cells.

### Pull-down and co-localization of MPII and E-cadherin

MPII-E352A and FRA3-E349A (2 µg each) were added to the HT29 cell lysate aliquots (2 mg total protein each). The protease constructs were then immunoprecipitated from the lysate samples using the FLAG antibody beads. The precipitates were next analyzed by Western blotting with the E-cadherin antibody and also with CD44 antibody (a control). The results indicated that, because of the interactions of MPII (but not FRA3) with E-cadherin, significant amounts of the latter were co-precipitated jointly with MPII and then readily detected by Western blotting (Fig. 6B). In turn, the tests with another abundant cell receptor (e.g., CD44) were negative, suggesting that MPII-E352A selectively pulled-down E-cadherin, but not CD44, from cell lysates.

To additionally confirm the direct binding of MPII to cellular E-cadherin, HT29 cells were co-incubated with the MPII-E352A mutant and then stained using the E-cadherin and Hisx6 antibodies. There was a clear co-localization of E-cadherin with the MPII-FLAG immunoreactivity in the cell-to-cell junction regions in HT29 cells (Fig. 6C).

## Discussion

The enterotoxigenic *B. fragilis* strains are known to produce a proteolytic toxin that may trigger colon cancer in mice and may also do so in humans. The toxin is a metalloprotease named BFT (*B. fragilis* toxin or FRA) that drives pathways known to be linked to inflammation and colorectal cancers [2], [22]. Three known highly homologous isoforms of FRA (FRA1, FRA2 and FRA3, which differ by only a few substitutions) are encoded by a pathogenicity island. There is, however, the second metalloproteinase, MPII, that is also encoded by the pathogenicity island in the *B. fragilis* pathogen and that is only 25% homologous with FRA [11],[14]. FRA and MPII are counter-transcribed suggesting that these two proteinases play certain specialized functions in *B. fragilis* virulence [12].

MPII is capable of cleaving the basic motifs in the peptide and protein substrates and, as a result, the cleavage preferences of MPII resemble those of the furin-like proprotein convertases [11], [23]. As compared with MPII, FRA is a less selective proteinase and tolerates the presence of multiple hydrophobic, hydrophilic and charged residue types in multiple substrate positions. There is, however, a cleavage specificity overlap between MPII and FRA3 (a representative isoform of the FRA mini-family). The only *in vivo* demonstrated substrate of FRA is E-cadherin, a key component of cell-cell contacts [8], [13], [15]. FRA proteolysis of the E-cadherin ectodomain is dependent on biologically active FRA and is then followed by processing of the intracellular E-cadherin fragment by cellular presenilin-1/γ-secretase [11], [13], [15], [16]. It may be envisioned that these proteolytic events affect cellular adherens junctions and weaken cell-to-cell contacts, enabling *B. fragilis* to penetrate the intestinal epithelium. Regardless of its similar structural fold and its overlapping cleavage preferences relative to FRA, MPII, however, did not cause any significant cleavage of E-cadherin in our cell-based tests [11], [14].

Here, we provide evidence that, in contrast with FRA, MPII directly binds to adherens junction E-cadherin in a way that is similar to the routine ligand-receptor interactions. Thus, MPII, but not FRA3, directly and in a concentration-dependent fashion binds cell surfaces of the cells, which express high levels of E-cadherin. Reduction in the E-cadherin expression decreases the binding levels of MPII. The binding of MPII takes place *via* the catalytic domain and is most noticeable in the cell-to-cell junctions. Finally, MPII is immunoprecipitated from cell lysates jointly with E-cadherin, suggesting the presence of the direct interactions between MPII and E-cadherin. Taken together, our results provide compelling evidence that MPII associates with cellular E-cadherin. Furthermore, our most recent mutagenesis studies demonstrate that the C-terminal ten residue MPII segment that has an especially low homology level with FRA is essential for the binding of MPII with E-cadherin (to be published elsewhere). The nature of the cellular receptor of FRA, however, remains elusive [21].

In conclusion, the present study is focused on MPII and FRA, two secretory metalloproteinases encoded by the single pathogenicity island that is present in the enterotoxigenic *B. fragilis* strains. Regardless of their structural similarity and overlapping cleavage preferences, FRA, but not MPII, cleaves E-cadherin. In turn, MPII directly binds to, rather than cleaves, E-cadherin, its host cell surface receptor. As a result, our data suggest that FRA and MPII likely perform specialized proteolytic functions in the course of *B. fragilis* gastro-intestinal infections. It is now tempting to hypothesize that a cell surface-associated MPII-E-cadherin complex, rather than soluble MPII alone, performs a focused cleavage function in the adherens junction compartment in the intestinal epithelial cells and that the action of MPII is complementary to that of FRA in the virulence mechanism of *B. fragilis*.
