# Expression of Concern: Distinct Interactions with Cellular E-Cadherin of the Two Virulent Metalloproteinases Encoded by a *Bacteroides fragilis* Pathogenicity Island

**DOI:** 10.1371/journal.pone.0269292

**Published:** 2022-06-06

**Authors:** The PLOS ONE Editors

Following the publication of this article [1], concerns were raised regarding results presented in Figs 1 and 5. Specifically,

- In Fig 1B, when colour levels are adjusted, two rectangular areas in the background appear lighter than the rest of the background.
- In Fig 5B, brightfield images for FRA3 and FRA3-E349A appear to partially overlap.

**Fig 1:**
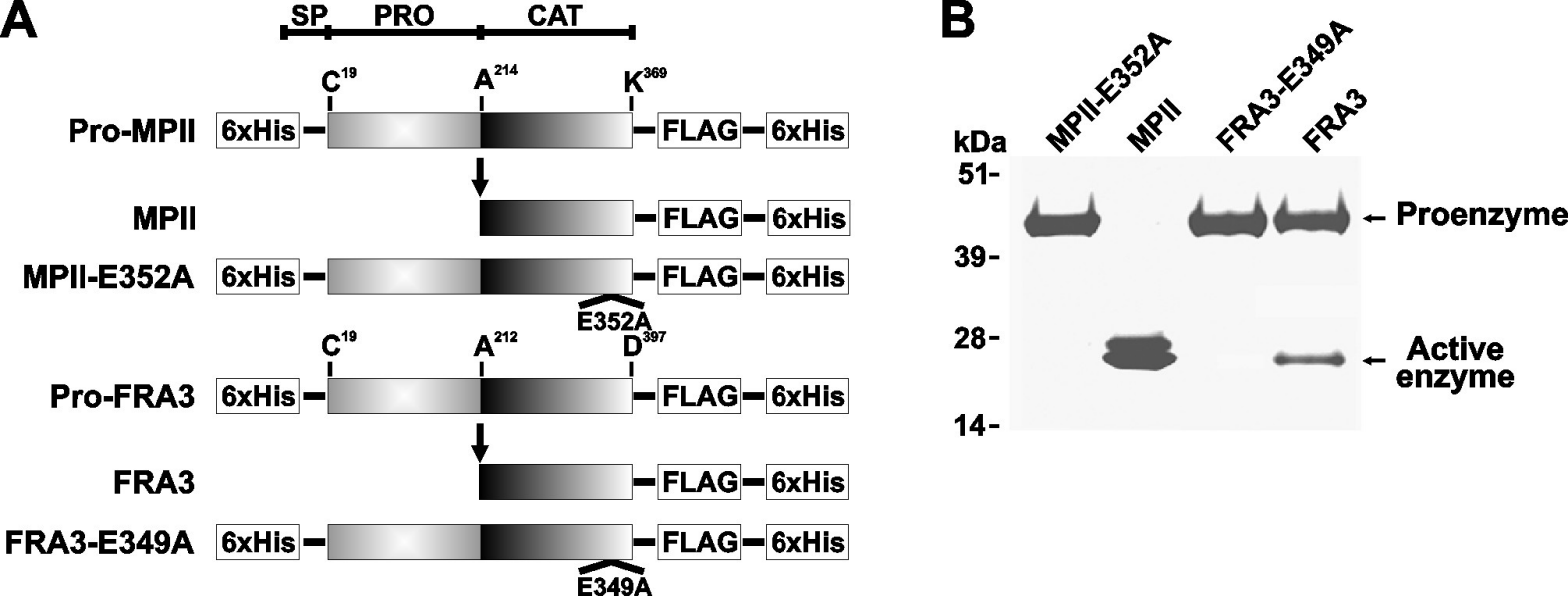
MPII and FRA3 recombinant constructs (alternative version provided by the corresponding author). **A**, the arrow indicates the position of self-conversion of the proenzyme into the fully active enzyme. The constructs were tagged with the Hisx6 and FLAG tags. E349A and E352A indicate the base substitution sites that transform the wild-type FRA3 and MPII into the catalytically inactive constructs of FRA3 and MPII, respectively. As a result of these inactivating mutations, Ala substitutes for the essential active site Glu residue. SP, signal peptide; PRO, prodomain; CAT, catalytic domain. **B**, SDS-gel electrophoresis of the purified MPII and FRA3 constructs followed by Coomassie staining.

**Fig 5:**
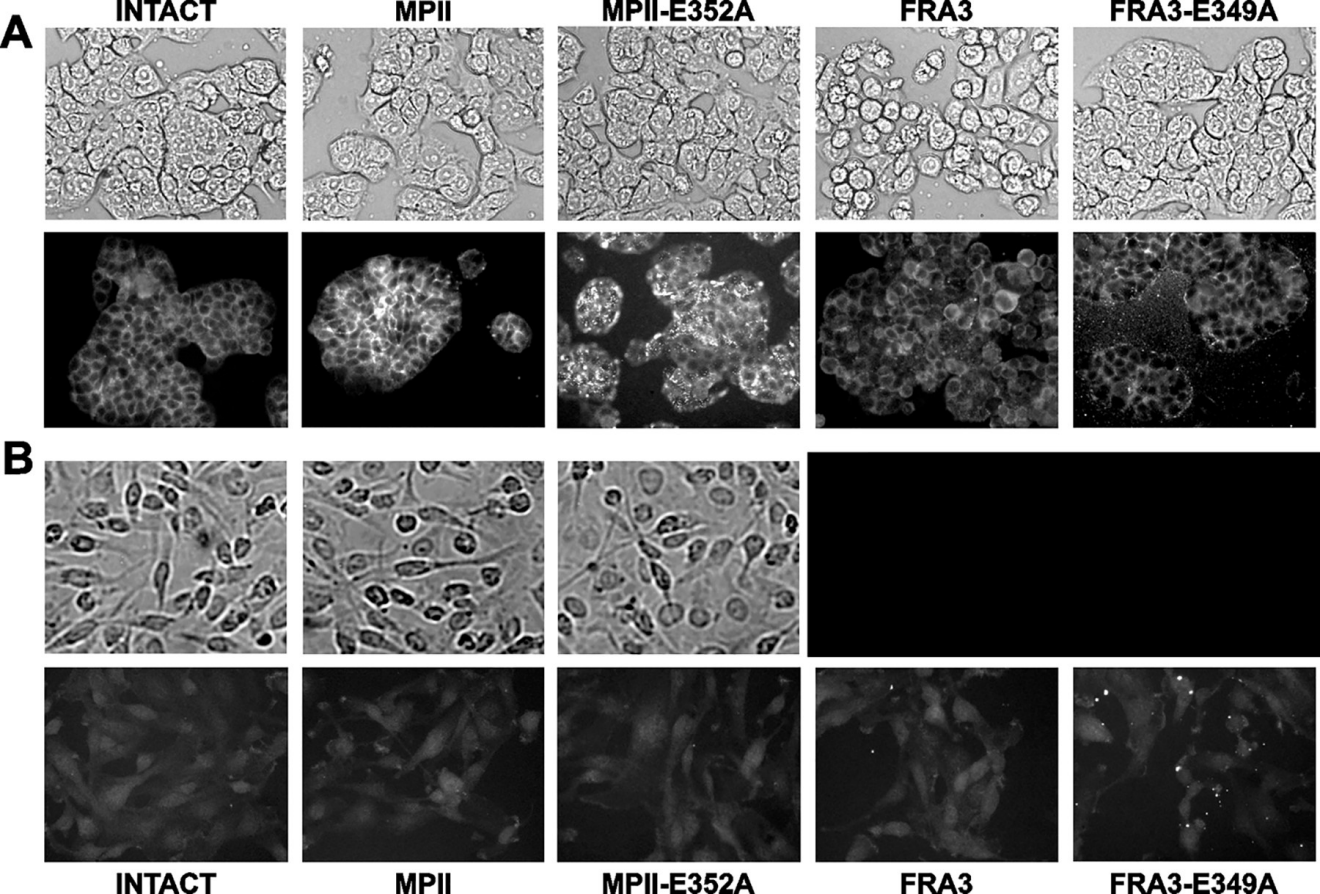
Immunostaining of cell-bound MPII. **A**, HT29 cells. **B**, U251 cells. Cells were left intact or incubated for 3 h at 37°C with MPII, MPII-E352A, FRA3 or FRA3-E349A (5 µg/ml, each). Cells were fixed, permeabilized and stained with the FLAG M2 antibody. Cell morphology was also observed using a bright-field microscope. The FRA3 and FRA3-E349A panels in Fig 5B are redacted as they partially overlapped and it was not possible to confirm which panel accurately presented the described results.

The corresponding author stated that they no longer have access to the raw data underlying the above figures or the other figures of this article. As the raw data are no longer available, the article is not in compliance with *PLOS ONE*’s Data Availability policy that was in place at the time of the article’s submission.

The corresponding author stated that the rectangular areas in the background of Fig 1B may have occurred in the course of figure preparation, and they provided an alternative version of Fig 1 (included with this notice). The rectangular areas are also visible in the background of the alternative version of Fig 1B when colour levels are adjusted. The editors remain concerned about this figure, and consider the issue unresolved in the absence of raw data.

The corresponding author acknowledged that the brightfield images for FRA3 and FRA3-E349A partially overlap, and stated that this occurred due to an error in figure preparation. They were unable to provide replacements for the overlapping panels, and in the absence of raw data, it is not possible to confirm which panel accurately presents the described results. Therefore, an updated version of Fig 5 is provided here in which both overlapping panels have been redacted. The corresponding author stated that the findings presented in Fig 5B are supported by results presented in Figs 1B, 2C, 5A and 6A.

The *PLOS ONE* Editors issue this Expression of Concern to notify readers of the above image concerns which are not fully resolved, and of the unavailability of raw data underlying the results in this article.
